# Identification of multiple interacting alleles conferring low glycerol and high ethanol yield in *Saccharomyces cerevisiae* ethanolic fermentation

**DOI:** 10.1186/1754-6834-6-87

**Published:** 2013-06-11

**Authors:** Georg Hubmann, Lotte Mathé, Maria R Foulquié-Moreno, Jorge Duitama, Elke Nevoigt, Johan M Thevelein

**Affiliations:** 1 Laboratory of Molecular Cell Biology, Institute of Botany and Microbiology, KU Leuven, Kasteelpark Arenberg 31, Leuven-Heverlee, Flanders B-3001, Belgium; 2 Department of Molecular Microbiology, VIB, Kasteelpark Arenberg 31, Leuven-Heverlee, Flanders B-3001, Belgium; 3 Agrobiodiversity reasearch area, International Center for Tropical Agriculture (CIAT), A.A. 6713, Cali, Colombia; 4 School of Engineering and Science, Jacobs University Bremen gGmbH, Campus Ring 1, Bremen 28759, Germany

**Keywords:** Complex trait, QTL analysis, Minor QTL, Causative gene, Glycerol yield, *Saccharomyces cerevisiae*, Pooled-segregant analysis, Backcross, Reverse metabolic engineering, Epistasis

## Abstract

**Background:**

Genetic engineering of industrial microorganisms often suffers from undesirable side effects on essential functions. Reverse engineering is an alternative strategy to improve multifactorial traits like low glycerol/high ethanol yield in yeast fermentation. Previous rational engineering of this trait always affected essential functions like growth and stress tolerance. We have screened *Saccharomyces cerevisiae* biodiversity for specific alleles causing lower glycerol/higher ethanol yield, assuming higher compatibility with normal cellular functionality. Previous work identified *ssk1*^*E330N…K356N*^ as causative allele in strain CBS6412, which displayed the lowest glycerol/ethanol ratio.

**Results:**

We have now identified a unique segregant, 26B, that shows similar low glycerol/high ethanol production as the superior parent, but lacks the *ssk1*^*E330N…K356N*^ allele. Using segregants from the backcross of 26B with the inferior parent strain, we applied pooled-segregant whole-genome sequence analysis and identified three minor quantitative trait loci (QTLs) linked to low glycerol/high ethanol production. Within these QTLs, we identified three novel alleles of known regulatory and structural genes of glycerol metabolism, *smp1*^*R110Q,P269Q*^, *hot1*^*P107S,H274Y*^ and *gpd1*^*L164P*^ as causative genes. All three genes separately caused a significant drop in the glycerol/ethanol production ratio, while *gpd1*^*L164P*^ appeared to be epistatically suppressed by other alleles in the superior parent. The order of potency in reducing the glycerol/ethanol ratio of the three alleles was: *gpd1*^*L164P*^ > *hot1*^*P107S,H274Y*^ ≥ *smp1*^*R110Q,P269Q*^.

**Conclusions:**

Our results show that natural yeast strains harbor multiple specific alleles of genes controlling essential functions, that are apparently compatible with survival in the natural environment. These newly identified alleles can be used as gene tools for engineering industrial yeast strains with multiple subtle changes, minimizing the risk of negatively affecting other essential functions. The gene tools act at the transcriptional, regulatory or structural gene level, distributing the impact over multiple targets and thus further minimizing possible side-effects. In addition, the results suggest polygenic analysis of complex traits as a promising new avenue to identify novel components involved in cellular functions, including those important in industrial applications.

## Introduction

Rational genetic modification of industrial microorganisms using targeted deletion and/or overexpression of structural or regulatory genes very often results in undesirable side effects on other essential functions [1-8]. This has severely compromised the development of new superior industrial microorganisms. Glycerol yield in *Saccharomyces cerevisiae* is a complex genetic trait with great industrial importance. Low glycerol production is essential for maximal yield in bioethanol production [5,9,10] while a high glycerol yield and a reduced ethanol yield are positive traits in wine production [11-14]. Rational genetic engineering of glycerol yield by modification of the main structural gene, *GPD1*, encoding glycerol 3-phosphate dehydrogenase (GPDH), the rate limiting enzyme of the glycerol biosynthesis pathway, has not been successful in obtaining appropriate industrial yeast strains with a modified glycerol/ethanol ratio due to the negative side-effects on other phenotypic traits. Deletion and even reduced expression of *GPD1* lowers growth and fermentation rates [2-5] while overexpression causes redox imbalance and overproduction of acetate and other by-products [1]. Genetic analysis of natural *S. cerevisiae* strains exhibiting an inherent glycerol yield significantly different from that of the industrial yeast strains to be improved, offers a promising strategy to identify mutant alleles suitable as gene tools for engineering glycerol production to obtain lower or higher yield, without causing negative side-effects on other essential traits.

Glycerol production is of great physiological importance in *S. cerevisiae*. Besides CO_2_, glycerol is the main quantitatively important side-product of yeast ethanolic fermentation. It is synthesized from dihydroxyacetone phosphate (DHAP) by the consecutive action of glycerol 3-phosphate dehydrogenase, encoded by the isogenes *GPD1* and *GPD2*, and glycerol 3-phosphate phosphatase, encoded by the isogenes *GPP1* and *GPP2*[3,15-17]. The first step of glycerol formation is accompanied by the oxidation of NADH + H^+^ to NAD^+^. One important cellular function of glycerol formation is to regenerate NAD^+^ during anaerobic growth in order to maintain the cytosolic redox balance. This is crucial since intermediates from the lower part of glycolysis are withdrawn for multiple biosynthetic pathways. As a result, some of the NADH + H^+^ generated upstream in glycolysis cannot be regenerated through ethanol formation. Glycerol formation is also essential during osmostress where it serves as the major compatible osmolyte. The high osmolarity glycerol (HOG) pathway plays an important role in the stimulation of glycerol production during osmostress and has been elucidated and characterised in great detail [18]. It involves osmosensing proteins at the level of the plasma membrane, a MAP kinase signaling pathway and transcription factors and other target proteins, that regulate glycerol production and intracellular accumulation. Both physiological functions of glycerol formation, i.e. redox balancing and coping with osmostress, are important during industrial ethanol production due to anaerobic conditions and high sugar concentrations (osmotic pressure) at the beginning of the process.

Glycerol production is a complex quantitative trait and glycerol yield was shown to be highly variable within the species *S. cerevisiae*[19]. This intraspecies diversity provides a promising starting point to understand and engineer the genetic basis for a low glycerol yield in industrial strains of *S. cerevisiae*. Pooled-segregant whole-genome sequencing has been developed as an efficient method to map quantitative trait loci (QTLs) involved in complex traits [20-24] and reciprocal hemizygosity analysis to identify the causative genes in the QTLs [25]. Random inbreeding of segregants combined with phenotypic selection can be used to increase the recombination frequency, making the QTLs smaller and thus facilitating identification of the causative genes [23]. In this case, millions of segregants were used and submitted to phenotypic selection, which enabled identification of many minor QTLs and the causative genes within these QTLs. However, this strategy only works for selectable traits. Most industrially relevant complex traits are non-selectable and phenotyping such large numbers of segregants is not feasible in practice. Hence, it remains highly important to develop alternative methodologies for analyzing minor QTLs in an efficient and reliable way that are applicable to hundreds instead of thousands or millions of segregants. Reliable identification and analysis of minor QTLs and their causative genes is challenging because they show only weak linkage and their contribution to the phenotype is easily overruled by major causative genes and/or can be replaced by other minor causative genes. One strategy to identify minor QTLs consists of replacing in the superior parent the causative alleles identified in major QTLs by the corresponding inferior alleles from the control parent strain. The resulting downgraded superior strain is then crossed again with the control parent strain [26]. Similarly, major QTLs were eliminated by targeted backcrossing to reveal minor QTLs [27,28]. A disadvantage of this strategy is that the phenotypic difference between the parent strains becomes less obvious and that therefore larger numbers of segregants may be required for reliable phenotyping and QTL mapping. Another strategy to identify minor QTLs is to increase the stringency of phenotypic screening. Swinnen et al. [20] showed that selection of yeast segregants tolerant to 17% ethanol versus 16% ethanol, strengthened the linkage of several minor QTLs, facilitating their further analysis. However, this methodology also requires higher numbers of segregants to be phenotyped.

In the present paper, we present a novel approach to identify minor QTLs, which does not suffer from the drawbacks that the phenotypic difference between the parent strains becomes smaller or that the number of segregants required for the screening increases. We have screened the F1 segregants for the combined presence of the superior trait and absence of a major causative gene previously identified. Only one such segregant could be identified, which was then used in a backcross with the inferior parent strain. We demonstrate that the segregants from this cross can be successfully used to map minor QTLs, of which we validated several by identifying the causative genes. This approach was applied to the non-selectable phenotype of low glycerol/high ethanol production in yeast fermentation, for which we previously identified *ssk1*^*E330N…K356N*^ as a major causative allele [19]. A backcross with the single segregant displaying low glycerol yield and lacking the *ssk1*^*E330N…K356N*^ allele led to the identification of three new minor QTLs, in which we identified as causative genes specific alleles of known genes in glycerol metabolism and its regulation, each causing a reduction of glycerol yield.

## Results

### Selection of a rare segregant displaying the trait of low glycerol/high ethanol yield and lacking the major causative allele ssk1^E330N…K356N^

**Figure 1:**
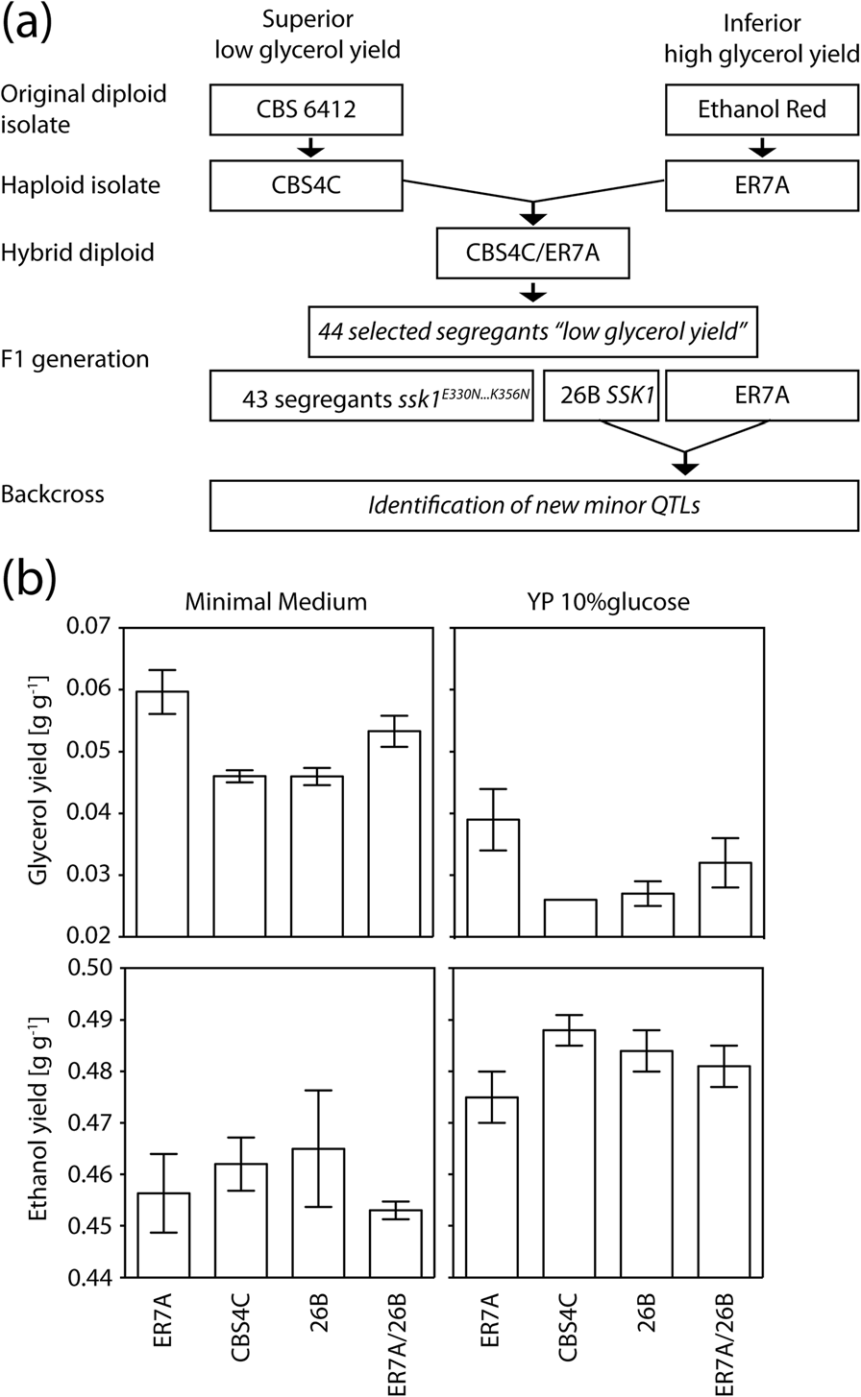
**Phenotypes of the parental strains ER7A and CBS4C and the segregant 26B.** (**a**) Scheme of the crossings to map mutations linked to the low glycerol yield phenotype. The initial parental cross of ER7A and CBS4C resulted in the segregant 26B with a low glycerol phenotype but without the *ssk1*^*E330N…K356N*^ allele. The 26B segregant was crossed back with the inferior parent ER7A to find other linked mutations. (**b**) Glycerol and ethanol yield (on glucose) obtained in minimal medium with 5% glucose and in YP 10% glucose for the parental strains, ER7A and CBS4C, the segregant 26B, and the hybrid diploid 26B/ER7A. Three independent fermentations were performed with each strain.

Previous work has identified the *S. cerevisiae* strain CBS6412 as a strain with an unusually low ratio of glycerol/ethanol yield and genetic analysis identified the *ssk1*^*E330N…K356N*^ allele as a major causative gene [19] (Figure 1a). In order to identify the minor QTLs and their causative genes responsible for determining this complex trait, we have first screened all superior segregants with a glycerol/ethanol ratio as low as the superior parent strain, for a segregant that lacked the *ssk1*^*E330N…K356N*^ allele. Among the 44 superior segregants available, only a single such segregant, 26B, was present. Its glycerol yield was equally low and its ethanol yield equally high as the superior parent strain CBS4C, both in minimal medium with 5% glucose and in rich YP medium with 10% glucose (Figure 1b). Hence, 26B showed the same phenotypic difference with the inferior parent strain ER7A as CBS4C (Figure 1b).

### Backcross of the unique superior segregant 26B with the inferior parent ER7A and screening for superior segregants

We next switched the mating type of 26B from Mat**α** to Mat**a** (see Materials and methods) and crossed the Mata 26B strain with the Mat**α** inferior parent strain, ER7A, which is a derivative of the industrial strain Ethanol Red, currently used worldwide in bioethanol production. The hybrid diploid ER7A/26B showed a glycerol/ethanol yield phenotype, which was intermediate between that of ER7A and 26B (Figure 1b). The hybrid was sporulated and 260 meiotic segregants were screened for low glycerol yield (and corresponding higher ethanol production) in 100 ml fermentations with YP 10% glucose. The parent strains 26B and ER7A, and the hybrid diploid, were used as controls in each batch of fermentations.

**Figure 2:**
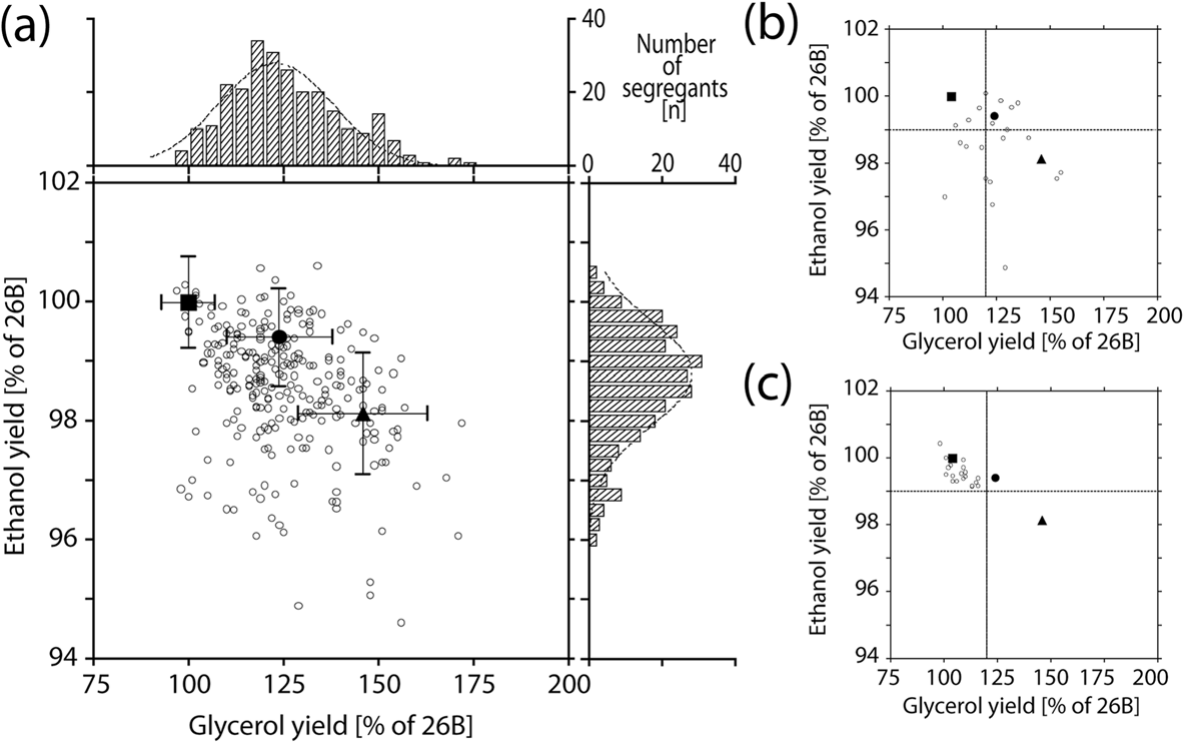
**Glycerol and ethanol yield (on glucose) in parental strains, hybrid diploid and segregants.** (**a**) Glycerol and ethanol yield (on glucose) in the parental strains, 26B (■) and ER7A (▲), the hybrid diploid strain 26B/ER7A (●) and in segregants of 26B/ER7A (○). For screening purposes, one fermentation was carried out for each strain in 100 ml YP with 10% glucose. Glycerol and ethanol yields of all segregants, ER7A and the diploid 26B/ER7A were related to the yield of 26B, which was set as 100%. (**b,c**) Distribution of the glycerol and ethanol yield (on glucose) in the unselected (**b**) and selected (**c**) segregant pool of 26B/ER7A. The criteria for selection of “low glycerol” segregants (<120% glycerol yield, >99% ethanol yield) are indicated with stippled lines. The values of the 22 selected segregants are the average of three replicates. These segregants were used for pooled-segregant whole-genome sequence analysis. The glycerol and ethanol yield of the parental strains, 26B and ER7A, and diploid 26B/ER7A are indicated as in (**a**).

Glycerol and ethanol yield of the segregants in each batch were normalized to those of 26B, which were set to 100%. ER7A and the diploid 26B/ER7A showed an average glycerol yield of 146% and 124% and a decreased ethanol yield of 98.1% and 99.4% (Figure 2a). The glycerol and ethanol yield of the segregants showed a Gaussian distribution, which extended over the range of the two parental strains. In the case of the lowest glycerol yield, this extension was only marginal. The population means of the glycerol yield (123%) and ethanol yield (98.8%) were close to those of the diploid 26B/ER7A. In general, glycerol and ethanol yield of the segregant population correlated inversely (as determined with a Pearson test), meaning that low glycerol yield was usually accompanied by high ethanol yield. Nearly all exceptions to this correlation were segregants with an unusually low ethanol yield that failed to show a correspondingly higher glycerol yield. To compose the pool of selected superior segregants, two cut-off criteria were defined, a glycerol yield lower than 120% of 26B and an ethanol yield higher than 99% of 26B. These cut-off criteria resulted in the selection of a set of 34 superior segregants. These were all retested in 100 ml fermentations with YP 10% glucose and 22 segregants showed again a low glycerol yield combined with a correspondingly higher ethanol yield using the same cut-off criteria (Figure 2b). These 22 segregants were selected for QTL mapping with pooled-segregant whole-genome sequence analysis. A second pool with 22 randomly selected segregants was also subjected to pooled-segregant whole-genome sequence analysis and referred to as the unselected control pool (Figure 2b).

### Pooled-segregant whole-genome sequence analysis and QTL mapping

**Figure 3:**
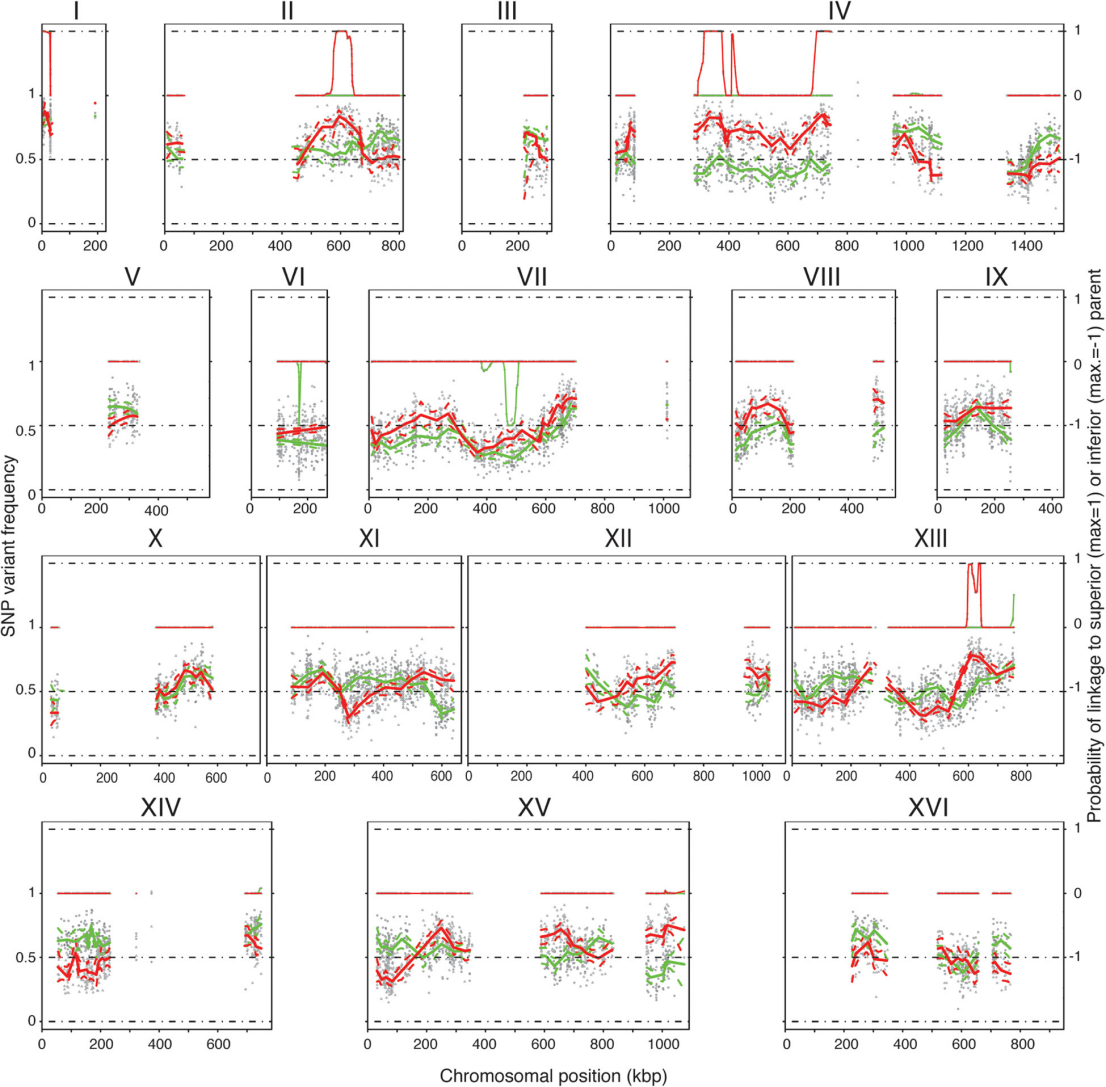
**Plots of SNP variant frequency versus chromosomal position and corresponding probability of linkage to the superior or inferior parent.** Plots of SNP variant frequency versus chromosomal position in all 16 yeast chromosomes for the selected (raw data: light grey triangles; smoothed data: red line) and unselected pool (raw data: light grey circles; smoothed data: green line). Significant upward deviations from the average of 0.5 indicate linkage to the superior parent 26B, while significant downward deviations indicate linkage to the inferior parent ER7A. The smoothed line was determined as described previously [20,29]. Linked regions were detected with EXPLoRA (Duitama et al. in preparation).

The genomic DNA of the selected and unselected pools, as well as the parent strain 26B, was extracted and submitted to custom sequence analysis using Illumina HiSeq 2000 technology (BGI, Hong Kong, China). The genome sequence of the parent strain ER7A has been determined in our previous study (data accession number SRA054394) [19]. Read mapping and single nucleotide polymorphism (SNP) filtering were carried out as described previously [20,29]. The SNP variant frequency was plotted against the SNP chromosomal position (Figure 3). Of the total number of 21,818 SNPs between CBS4C and ER7A, 5,596 SNPs of CBS4C were found back in 26B. These SNPs were used for mapping minor QTLs in the genomic areas that were not identical between 26B and ER7A. The other genomic areas were completely devoid of SNPs because they were identical between the 26B and ER7A parents (white gaps in Figure 3). The scattered raw SNP variant frequencies were smoothened and a confidence interval was calculated, as previously described [20,29]. The Hidden Markow Model, EXPloRA (see Materials and methods) was used to evaluate whether candidate regions showed significant linkage to the low glycerol phenotype. EXPloRA indicated six significant QTLs: on chr. I (3859–11045), chr. II (584232–619637), chr. IV (316389–375978 and 696486–748140), and chr. XIII (600902–610995 and 634582–640415) for the selected segregants pool.

The locus on chr. I was present in both the selected and unselected pool and was thus likely linked to an inadvertently selected trait, such as sporulation capacity or spore viability. It was excluded from further analysis. EXPloRA also reported two significantly linked loci on chr. VI (169586–170209) and chr. VII (472620–493523) for the unselected pool. Both loci were linked to the inferior parent, ER7A. For the region on chr. VII, the linked locus with the inferior parent genome was also present in the selected pool. Both loci likely represent linkage to inadvertently selected traits, such as sporulation capacity or spore viability. It is unclear why the locus on chr. VI was only present in the unselected pool. Since both loci were not linked to the low glycerol phenotype they were not investigated further.

The locus on chr. II was interesting since it also appeared in the previous mapping with the two original parents, CBS4C and ER7A, but in that case it was not pronounced enough to be significant [19]. The mapping with the backcross has now confirmed the relevance of this locus. On chr. IV and XIII, two new QTLs with a significant linkage to the low glycerol/high ethanol yield phenotype were detected. These QTLs were not present in our previous mapping with the original parent strains CBS4C and ER7A.

**Figure 4:**
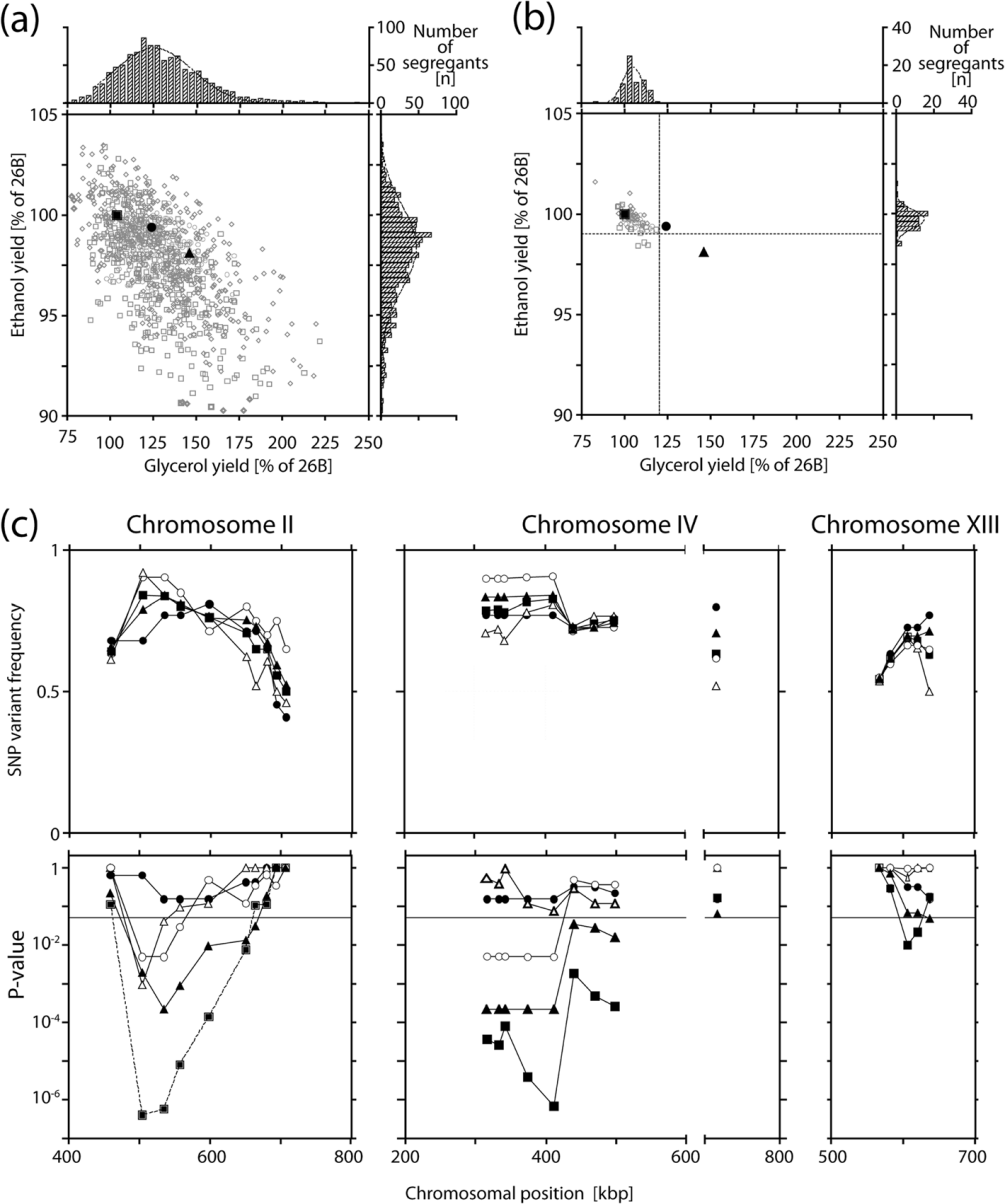
**Linkage analysis of QTLs on chr. II, IV and XIII with different groups of segregants.** (**a**) Glycerol and ethanol yield (on glucose) of the parental strains, 26B (■) and ER7A (▲), and the hybrid diploid strain 26B/ER7A (●). Glycerol and ethanol yield of the first isolated F1 segregants from 26B/ER7A (○), of the additional F1 segregants (□) and of the F5 segregants (◊). For screening purposes, one fermentation was carried out in 5 ml YP 10% glucose. Glycerol and ethanol yield of all segregants, ER7A and the diploid 26B/ER7A were related to the yield of 26B, which was set as 100% (**b**) Segregants were selected for low glycerol (<120% glycerol yield, stippled line) and high ethanol (>99% ethanol yield, stippled line) yield (on glucose) after each round of screening, resulting in the following segregant groups: 22 F1 segregants used for pooled-segregant whole-genome sequence analysis (○), 22 additional selected F1 segregants (□), and 26 F5 segregants (◊). These segregants were reconfirmed in 100 ml YP 10% glucose. Values for glycerol and ethanol yield are the average of three replicates. (**c**) SNP variant frequency (top) and respective P-value (bottom) were determined by allele-specific PCR in individual segregants of the sequenced selected pool (●), additional F1 selected pool (○), the total F1 selection of 44 (▲), the selection of F5 segregants (△), and the total selection of all 70 segregants (■) to fine-map the QTLs on chr. II, IV and XIII, which were detected with EXPloRA. The statistical confidence line (for P-value ≤ 0.05) is indicated with a stippled line.

All QTLs with a significant link to the phenotype under study, i.e. those on chr. II, IV and XIII, were further investigated in detail. Selected SNPs within the respective QTLs were scored in the 22 individual superior segregants to determine precisely the SNP variant frequency and the statistical significance of the linkage. Using the binomial test previously described [20,29] none of the three loci was found to be significantly linked to the genome of the superior parent strain 26B with the low number of superior segregants available. Therefore, we isolated 400 additional F1 segregants of the diploid 26B/ER7A and screened them for low glycerol/high ethanol production. In addition, we performed four rounds of random inbreeding (mating and sporulation) with all F1 segregants from the diploid 26B/ER7A to increase the recombination frequency [23] and subsequently also evaluated 400 F5 segregants in small-scale fermentations for glycerol/ethanol yield. The results for the 400 F1 and 400 F5 segregants are shown in Figure 4a. The glycerol and ethanol yields are again expressed as percentage of that of the superior parent strain 26B. There was again a clear inverse correlation between glycerol and ethanol yield. From the 800 segregants, we selected in total 48 superior segregants, i.e. 22 F1 segregants and 26 F5 segregants (Figure 4b).

We next scored selected SNPs in the putative QTLs on chr. II, IV and XIII in the 22 additionally selected F1 segregants and the 26 selected F5 segregants. Next, we determined the SNP variant frequency and the corresponding P-value, as described previously [20,29], for the following groups of segregants: the 22 initially selected segregants of the sequenced pool, the 22 additionally selected F1 segregants, the total of 44 selected F1 segregants, the 26 selected F5 segregants and the total of 70 selected F1 and F5 segregants. They are shown in Figure 4c. By increasing the number of superior segregants, we were now able to demonstrate significant linkage (P-value < 0.05) to the genome of the superior parent strain 26B for the three QTLs under study. For the QTLs on chr. II and IV the linkage was very strong, while for the QTL on chr. XIII it was still weak, but significant. In contrast, the second region on chr. IV did not show any significant linkage with none of the pools.

### Identification of causative genes in the QTLs on chr. II, IV and XIII

For further analysis, we first selected three potential candidate genes within the three QTLs, based on their known function in glycerol metabolism. *SMP1*, which is located in the QTL on chr. II (594,864 to 593,506 bp), encodes a putative transcription factor involved in regulating glycerol production during the response to osmostress [30]. The gene is located in the chromosomal region from 584,232 to 619,637 bp, which was predicted as most significant by the EXPloRA model. The 26B *SMP1* allele has two point mutations within its coding sequence, which are changing the primary protein sequence at position 110 from arginine to glutamine and at position 269 from proline to glutamine. Hence, we have named this allele *smp1*^*R110Q,P269Q*^.

The SNP with the highest linkage within the QTL found on chr. IV, was located at position 411,831 bp (Figure 4c), which is within the open reading frame of *GPD1* (411,825 to 413,000 bp). This is the structural gene for the NAD^+^-dependent cytosolic GPDH [15,16]. This enzyme catalyzes the conversion of DHAP to glycerol 3-phosphate through the oxidation of NADH and has been shown to be the rate-controlling step in glycerol formation [1,16]. The *GPD1* allele of 26B harbors a point mutation, changing leucine at position 164 into proline. This mutation was found before (DDBJ database data, accession number AY598965). The *GPD1* allele of 26B was named *gpd1*^*L164P*^.

The SNP with the highest linkage within the QTL found on chr. XIII was located at position 606,166 bp (Figure 4c), which is within the open reading frame of *HOT1* (605,981 to 608,140 bp). *HOT1* encodes a transcription factor required for the response to osmotic stress of glycerol biosynthetic genes, including *GPD1*, and other HOG-pathway regulated genes [31,32]. The 26B *HOT1* allele contains two non-synonymous point mutations, changing proline to serine at position 107 and histidine to tyrosine at position 274. We have named the *HOT1* allele of 26B, *hot1*^*P107S,H274Y*^.

**Figure 5:**
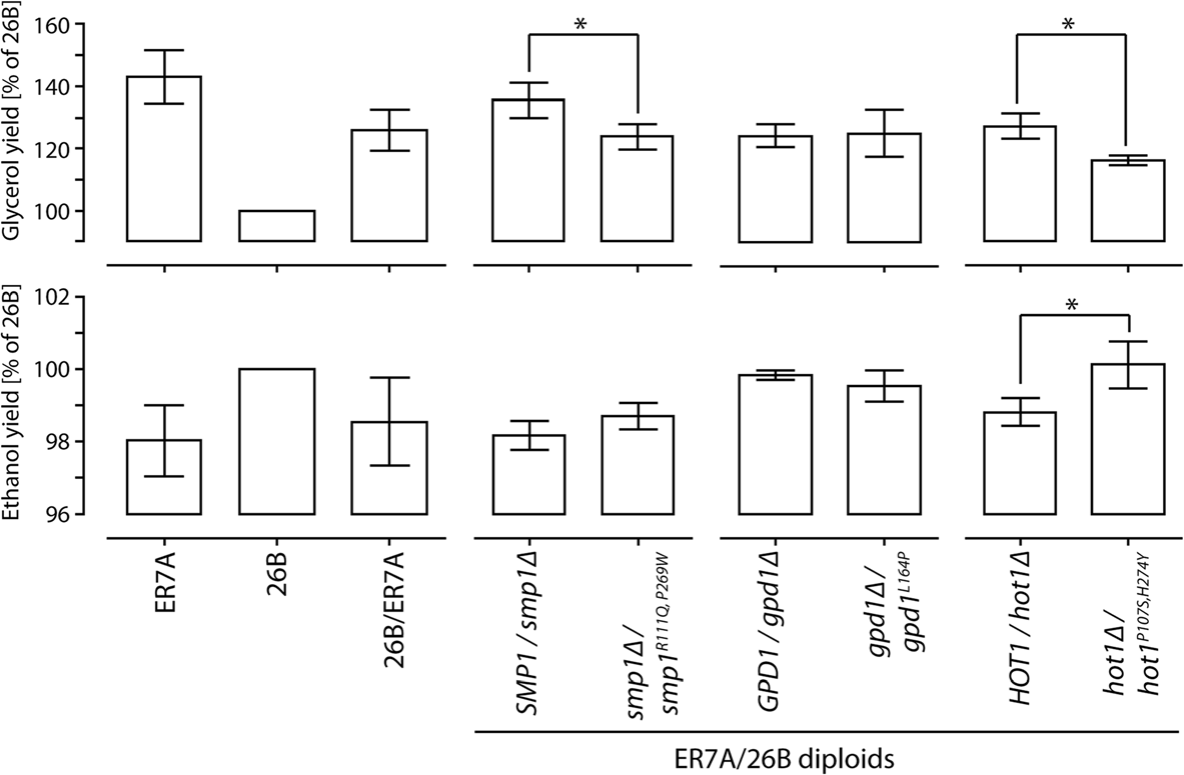
**Reciprocal hemizygosity analysis (RHA). RHA for the candidate genes, *SMP1 *(chr. II), *GPD1 *(chr. IV), and *HOT1 *(chr. XIII) to evaluate them as causative genes in the QTLs.** For RHA, diploid strains were constructed with either the deletion of the ER7A allele or the deletion of the 26B allele. Glycerol and ethanol yield (on glucose) of the two hemizygous diploid strains were related to the parental strain 26B. The Student *t*-test was used to confirm significant differences in glycerol and ethanol yield for the two diploids and is indicated with *. Each strain construct was tested in triplicate.

We first investigated the effect of *smp1*^*R110Q,P269Q*^, *gpd1*^*L164P*^ and *hot1*^*P107S,H274Y*^on the low glycerol/high ethanol phenotype using reciprocal hemizygosity analysis (RHA) [25]. For that purpose, we constructed for each gene a pair of hemizygous diploid 26B/ER7A hybrid strains, in which each pair contained a single copy of the superior or the inferior allele of *SMP1, GPD1* or *HOT1*, respectively, while the other copy of the gene was deleted. The three pairs of hemizygous diploids were tested in the same 100 ml YP 10% glucose fermentations as previously used for the screening. The parent strains 26B and ER7A and the hybrid diploid 26B/ER7A were added as controls. The glycerol and ethanol yields were again expressed as percentage of those of 26B, which were set at 100%. The significance of any differences between the strains was evaluated using a two-tailed unpaired *t*-test with a P-value < 0.05 considered to indicate a significant difference. The results of the RHA are shown in Figure 5. They indicate that both *smp1*^*R110Q,P269Q*^ and *hot1*^*P107S,H274Y*^, but not *gpd1*^*L164P*^, derived from the superior parent 26B cause a significant drop in the glycerol/ethanol ratio compared to the alleles of the inferior parent strain ER7A. For *smp1*^*R110Q,P269Q*^ only the reduction in glycerol, and not the increase in ethanol, was significant with the P-value < 0.05 used. These results indicate that *smp1*^*R110Q,P269Q*^ is probably a causative gene in the QTL on chr. II. They do not exclude that the QTL may contain a second causative gene, especially since *smp1*^*R110Q,P269Q*^ is not located in the region with the strongest linkage (lowest P-value).

The RHA with the *GPD1* alleles failed to show any difference both for glycerol and ethanol production (Figure 5). Hence, the superior character of the *gpd1*^*L164P*^ allele could not be confirmed with RHA. This is remarkable because the SNP with the strongest linkage (lowest P-value) in the QTL on chr. IV was located in the open reading frame of *GPD1* and showed very strong linkage to the low glycerol/high ethanol phenotype. The *hot1*^*P107S,H274Y*^ allele of the superior strain 26B, in contrast, caused a reduction in glycerol and an increase in ethanol production, and both changes were significant (P-value < 0.05) (Figure 5). Hence, these results indicate that *hot1*^*P107S,H274Y*^ is a causative allele in the QTL on chr. XIII and because it contains the SNP with the strongest linkage (lowest P-value), it is likely the main causative allele in this QTL.

The glycerol yield for the inferior parent ER7A and the diploid 26B/ER7A were on average 143% and 126% of the 26B yield (Figure 5). Ethanol yield of both strains was correspondingly reduced to 98% of the 26B yield. Clearly, the *smp1*^*R110Q,P269Q*^ and *hot1*^*P107S,H274Y*^ alleles can only be responsible for part of the difference in the glycerol/ethanol ratio between the parent strains. The same was found previously for the *ssk1*^*E330N…K356N*^ allele [19]. This confirms that the glycerol/ethanol ratio in yeast fermentation is a true polygenic, complex trait, determined by an interplay of multiple mutant genes.

### Expression of the *gpd1*^*L164P*^ allele from 26B in haploid *gpd1*∆ strains reveals its superior character

**Figure 6:**
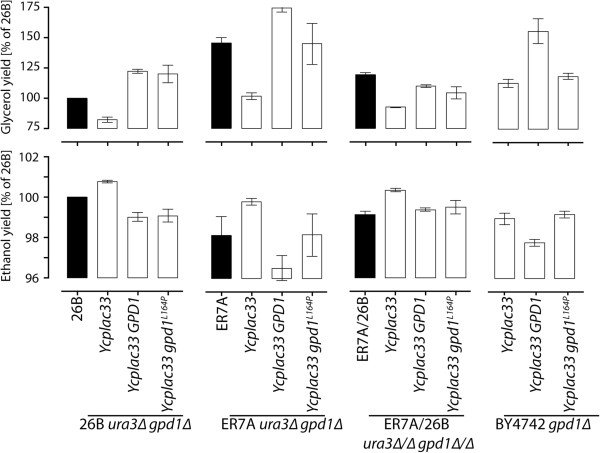
**Expression of *gpd1***^***L164P***^**-CBS4C and *GPD1*-ER7A in segregant 26B, ER7A, the diploid 26B/ER7A and BY4742.** Glycerol and ethanol yield (on glucose) in the *gpd1Δ* strains, 26B, ER7A, 26B/ER7A and BY4742, harboring the plasmids YCplac33, YCplac33 GPD1-ER7A, and YCplac33 gpd1^L164P^-CBS4C. Fermentations were carried out in 100 ml YP 10% glucose. Each strain construct was tested in triplicate. Glycerol and ethanol yield of the strains were related to the yield of 26B, which was set at 100%. In the BY4742 and ER7A backgrounds, which lack the *smp1*^*R110Q,P269Q*^ and *hot1*^*P107S,H274Y*^ alleles, the *gpd1*^*L164P*^ allele clearly reduced glycerol yield and concomitantly increased ethanol yield compared to the wild type *GPD1* allele. In the strains 26B and 26B/ER7A, which contain the *smp1*^*R110Q,P269Q*^ and *hot1*^*P107S,H274Y*^ alleles, the *gpd1*^*L164P*^ allele resulted in a similar glycerol yield as the wild type *GPD1* allele.

Several explanations could account for the failure to confirm the superior character of the *gpd1*^*L164P*^ allele from 26B in the RHA test. A closely located gene may be the real causative gene, the *gpd1*^*L164P*^ allele may be effective only in a haploid genetic background or the effect of the *gpd1*^*L164P*^ allele may be suppressed through epistasis by one or both of the other two superior alleles, *smp1*^*R110Q,P269Q*^ and *hot1*^*P107S,H274Y*^. To distinguish between these possibilities, we amplified the *gpd1*^*L164P*^ allele from strain CBS4C and the *GPD1* allele from strain ER7A by PCR (410,523 to 413,479 bp, including promotor, ORF and terminator). The PCR fragment was ligated in the centromeric plasmid YCplac33, resulting in plasmids YCplac33/gpd1^*L164P*^-CBS4C and YCplac33/GPD1-ER7A. Both plasmids were transformed into *gpd1∆* strains of the two parents 26B and ER7A, the hybrid diploid 26B/ER7A and the lab strain BY4742 [33,34]. All strains were tested in 100ml fermentations with YP 10% glucose. Glycerol and ethanol yields were determined after 120 h of fermentation. The results are shown in Figure 6.

When the *gpd1*^*L164P*^*-CBS4C* allele or the *GPD1-ER7A* allele were expressed in the *gpd1∆* strains of the superior parent 26B or the hybrid diploid 26B/ER7A, the increase in glycerol production and the decrease in ethanol production was the same for the two alleles. On the other hand, expression of the *gpd1*^*L164P*^*-CBS4C* allele in the *gpd1∆* strains of the inferior parent ER7A or the lab strain BY4742, enhanced glycerol production and reduced ethanol production significantly more than expression of the *GPD1-ER7A* allele. The latter shows that the *gpd1*^*L164P*^*-CBS4C* allele is superior compared to the *GPD1-ER7A* allele. The difference between the two alleles is apparently not dependent on the haploid or diploid background of the strain but seems to be related with the presence of the two other superior alleles, *smp1*^*R110Q,P269Q*^ and *hot1*^*P107S,H274Y*^. They are both present in the two strains, 26B and 26B/ER7A, in which *gpd1*^*L164P*^*-CBS4C* has no differential effect and absent in the two strains, ER7A and BY4742, in which *gpd1*^*L164P*^*-CBS4C* has a differential effect. Hence, the superior potency of *gpd1*^*L164P*^*-CBS4C* may be suppressed through epistasis by *smp1*^*R110Q,P269Q*^ and/or *hot1*^*P107S,H274Y*^. On the other hand, we cannot exclude that the effect of *gpd1*^*L164P*^*-CBS4C* is suppressed by one or more other mutant genes present in the superior parent 26B or the hybrid diploid 26B/ER7A.

**Figure 7:**
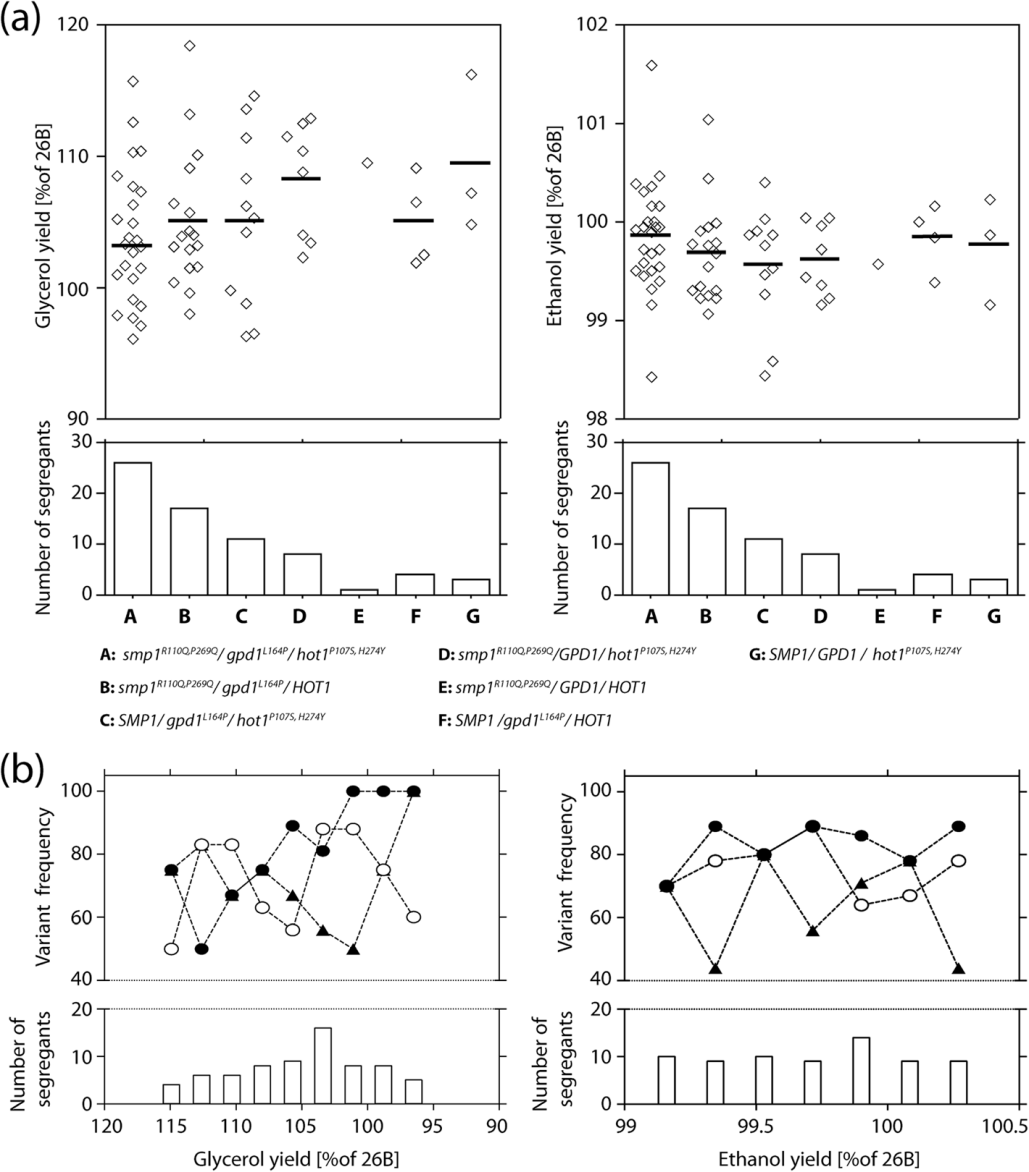
**Distribution of the *gpd1***^***L164P***^**, *hot1***^***P107S,H274Y ***^**and *smp1***^***R110Q,P269Q ***^**alleles in the selected low glycerol/high ethanol segregants.** (**a**) Glycerol and ethanol yield (on glucose) in segregants with different combinations of the superior alleles, *gpd1*^*L164P*^, *hot1*^*P107S,H274Y*^ and *smp1*^*R110Q,P269Q*^*,* in the selected segregant pool. The mean value of the glycerol and ethanol yield is indicated for each group. (**b**) Variant frequency of *gpd1*^*L164P*^ (●), *hot1*^*P107S,H274Y*^ (▲) and *smp1*^*R110Q,P269Q*^ (○) in the 70 selected segregants, which were categorized according to decreasing glycerol yield and increasing ethanol yield. Glycerol yield was divided into nine bins, each with a similar number of strains, starting from <96.5 and with a bin width of 2.3%. Accordingly, ethanol yield was divided into seven bins, each with a similar number of strains, starting from <99.16 and with a bin width of 0.185%. The number of segregants in each bin is indicated in the lower panel.

We have scored the final 70 superior segregants with a glycerol yield < 120% and an ethanol yield > 99% of that of the superior parent 26B, for the presence of the three causative alleles, *smp1*^*R110Q,P269Q*^, *gpd1*^*L164P*^ and *hot1*^*P107S,H274Y*^. The results are shown in Figure 7a. The largest group of superior segregants contained all three mutant alleles, followed by smaller groups with only two of the three mutant alleles and finally the three smallest groups with only one mutant allele. Hence, there was a clear correlation between the number of mutant alleles and low glycerol/high ethanol yield in this group of selected segregants. On the other hand, although there was a tendency for a lower mean glycerol/ethanol yield ratio with an increasing number of mutant alleles, the differences between the means of the different groups were small and the variation remained large and with the same range for the three largest categories.

We have also investigated a possible correlation between the different mutant alleles and the strength of the low glycerol/high ethanol phenotype. For that purpose, we determined the percentage of segregants with a specific mutant allele in sets of strains with a decreasing glycerol yield or an increasing ethanol yield. The results show that there is no preference between the different alleles in the strains with a higher glycerol yield, but in the strains with the lowest glycerol yield, the *gpd1*^*L164P*^ allele is preferentially present, followed by the *hot1*^*P107S,H274Y*^ allele, although this only holds for the category with the lowest glycerol yield (Figure 7b). Hence, the order of potency of the three alleles appears to be: *gpd1*^*L164P*^ > *hot1*^*P107S,H274Y*^ ≥ *smp1*^*R110Q,P269Q*^. There was no correlation between the variant frequency of the three alleles for high ethanol yield, indicating that other minor QTLs may affect ethanol yield independently from glycerol yield and act together with the currently identified alleles.

## Discussion

### Identification of superior alleles as gene tools for reduction of the glycerol/ethanol yield ratio

The goal of the present work was to investigate whether natural yeast strains may harbor specific alleles, e.g. in structural and/or regulatory genes of glycerol metabolism, that would allow reducing the glycerol yield and increasing the ethanol yield in yeast fermentation without causing negative side-effects on other essential functions. We successfully identified three mutant alleles, which separately and together reduce the glycerol yield in a subtle way without affecting, at least not in a conspicuous way, the overall rate and characteristics of the fermentation process. Combined with the previous discovery of the *ssk1*^*E330N…K356N*^ allele in strain CBS4C [19], this indicates that the original diploid parent strain CBS6412 contains at least four specific alleles causing reduced glycerol production and concomitantly higher ethanol production. This suggests that a low glycerol/ethanol yield ratio may have been advantageous for the survival of the *S. cerevisiae* strain CBS6412 in nature. Hence, we have now identified four mutant alleles acting at different levels, either transcriptional, regulatory or structural, in the biosynthesis of glycerol and its regulation, and that have been pre-filtered by natural selection and evolution for compatibility with survival in the natural environment. This spreads the reduction of glycerol yield not only over different target genes, each causing a subtle reduction, but also over different nodes in the cellular network and thus likely minimizes further the risk of negative side-effects.

For the phenotypic screening, we primarily selected on low glycerol yield. Although there was in general an inverse relationship between glycerol and ethanol yield, this was not true in all strains. Moreover, the variant frequency of the three mutant alleles for low glycerol yield and high ethanol yield (Figure 7b) did not match. In the segregants with the lowest glycerol yield, as opposed to those with higher glycerol yield, the *gpd1*^*L164P*^ allele was preferentially present, but this difference was not observed between segregants with higher and lower ethanol yield. This suggests that there are other factors that determine high ethanol yield independent of glycerol yield or that it is the combination of the mutant alleles that is required to enhance ethanol yield rather than the presence of specific alleles. The previously identified major *ssk1*^*E330N…K356N*^ allele, which was present in nearly all F1 segregants with low glycerol/ethanol ratio [19], might also have played a more important role in establishing high ethanol yield than the three currently identified minor causative alleles.

Our results therefore confirm that natural yeast strains harbor mutant alleles of the well known structural and regulatory genes identified in laboratory yeast strains that have been filtered by natural selection and evolution for compatibility with survival in the natural environment. The chance that these alleles exert significant negative effects on other essential functions of the yeast cells is probably not completely absent but at least minimized compared to drastic genetic modifications like gene deletion or overexpression. Screening of biodiversity for such specific alleles therefore appears to be a fruitful strategy to identify mutant alleles that can be used as specific gene tools for strain improvement by targeted genetic modification. A related example is the development of *S. cerevisiae* wine strains with a higher glycerol/lower ethanol production ratio [12-14]. Higher glycerol production is a preferred characteristic because it improves the mouthfeel of wine and lower ethanol production is a preferred characteristic as well because of the restrictions imposed on ethanol consumption for driving. Our results with the many segregants tested for glycerol/ethanol yield in fermentation has revealed many strains with a much higher glycerol and much lower ethanol yield than the parent strains (Figures 2, 4). This suggests that also for this beneficial trait specific alleles could be identified that would allow to improve this trait in a more subtle way. Possibly, this would allow to enhance glycerol production and reduce ethanol production without the dramatic increase in undesirable acetate production that was the result of classical genetic engineering of structural genes for GPDH [1].

### Identification of minor QTLs and causative genes

While identification of major QTLs has become straightforward with pooled-segregant whole-genome sequence analysis [19,20,22,23], identification of minor QTLs remains a major challenge. This is especially true for phenotypes that require a high workload for scoring and for which as a result only low numbers of selected segregants can be obtained to assemble the pool for whole-genome sequence analysis. In the present paper we have successfully established a novel approach for minor QTL identification. After mapping major QTLs and identification of the causative genes, the F1 segregants displaying the phenotype-of-interest are screened for absence of one or more superior alleles. As a result, these segregants should have all or most minor QTLs able to confer the phenotype-of-interest. This is not only because they are needed to confer the phenotype in the absence of the superior allele, but also because their effect is often suppressed by a major superior allele through epistasis. Hence, the use of a rare segregant displaying the trait-of-interest, might be advantageous in case of gene interference. To display the trait-of-interest, the segregant must have inherited a set of compatible mutant alleles, with any interfering mutations being absent. When such a rare F1 segregant is backcrossed with the inferior parent, the segregants from this cross displaying the phenotype-of-interest, should again contain all or most of these minor QTLs facilitating their mapping and identification of their causative allele. As shown in Figure 7a, several of these segregants contained only one of the three causative alleles and still displayed a low glycerol yield under the cut-off of 120%. This suggests that there are additional alleles present in these strains able to confer low glycerol yield and that a new backcross of such a segregant with the inferior parent may allow identification of additional alleles conferring low glycerol yield. In principle this approach could thus be repeated with each new generation of segregants. Previously, similar approaches have been used in which either F1 segregants were backcrossed to eliminate a major QTL [27] or major QTLs were fixed in one of the parents and the crossing repeated [26]. In these cases the parents displayed a reduced phenotypic difference, which may make the phenotyping and the stringency of selection in the next cross more cumbersome.

### Random coincidence versus linkage in small segregant populations

In small populations of segregants, random coincidence can easily cause falsely predicted QTLs, which are difficult to distinguish from QTLs with significant, but weak linkage [35]. In this case, the unselected pool is of little use because it usually does not contain at the same position the same false QTL caused by random coincidence. Higher stringency in QTL selection can eliminate false QTLs but also weakly linked true QTLs. An essential difference between a false QTL caused by random coincidence and a true QTL with weak linkage, is that the latter should be reproducible. Therefore, we screened three different pools of segregants resulting in three independent pools of small-size with segregants displaying low glycerol yield. This allowed us to distinguish the false QTLs on chr. IV (696486–748140) and chr. XIII (634582–640415) from the true, weakly linked QTLs on the same chromosomes, chr. IV (316389–375978) and chr. XIII (600902–610995). It is well known that a higher number of segregants increases the reliability of minor QTL detection. In previous work, many minor QTLs could be identified by using millions of segregants and a selectable phenotype [23]. However, most complex traits are not selectable and phenotypic screening of millions of segregants is not feasible for many traits. In these cases, reliable minor QTL identification remains a major challenge. In addition, the number of segregants that can be pooled for pooled-segregant whole genome sequence analysis is in principle unlimited, but in practice the useful number of segregants is limited by the average coverage in whole-genome sequencing. When the number of segregants exceeds the average coverage of sequencing, the surplus no longer enhances the reliability of mapping and is thus useless.

### Novel mutant alleles and possible epistatic interactions

Smp1 is a transcription factor, belonging to the MEF2 family, that regulates the expression of stress-responsive genes, such as *GPD1*. Its DNA binding domain is located at the amino acid residues 1–90 [36]. Upon osmotic stress, Smp1 is phosphorylated by Hog1, which physically interacts with its C-terminal domain. Four different phosphorylation sites were identified, i.e. Ser348, Ser357, Thr365, and Ser376, all located within a region coincident with the Hog1 binding domain. Phosphorylation of Smp1 is essential for its functioning, since an allele unable to be phosphorylated caused an impaired stress response [30]. The point mutations in the 26B allele, *smp1*^*R110Q,P269Q*^, are not located in the DNA or the Hog1 binding domain. However, the change of a proline to a glycine, close to the phosphorylation sites, might change Smp1 structure, thereby influencing its ability to be bound and/or phosphorylated by Hog1. The *smp1*^*R110Q,P269Q*^ allele is dominant since its expression in the hybrid 26B/ER7A diploid decreased glycerol yield and increased ethanol yield.

*GPD1* encodes NAD^+^-dependent cytosolic glycerol 3-phosphate dehydrogenase. It catalyzes the conversion of DHAP to glycerol 3-phosphate through the oxidation of NADH. The expression of *GPD1* is induced by the HOG pathway and it is essential for growth under high osmolarity [16] Possible domains for binding of NADH, H^+^ and DHAP have been predicted based on similarity with proteins with a comparable function [37]. The single point mutation present in the 26B allele, *gpd1*^*L164P*^, may be located in the putative NADH-binding domain, but the location of this domain is not well predicted. This mutation was found earlier and called a ‘natural variant’ (DDBJ database data, accession number AY598965). No linkage of *gpd1*^*L164P*^ with low glycerol yield was observed in RHA but its effect was revealed by expression in a *GPD1*-deficient mutant. The L164P mutation could reduce the intrinsic activity of the Gpd1 enzyme or its expression by lowering *GPD1* mRNA stability. Both possibilities would result in reduced glycerol production and thus explain the low glycerol yield of CBS6412. Both explanations are consistent with the lower GPDH activity that we measured in CBS4C (0.128 ± 0.027 U/mg protein) compared to ER7A (0.225 ± 0.053 U/mg protein).

The *gpd1*^*L164P*^ allele was apparently subject to epistatic suppression in the superior strains. In the BY4742 and ER7A backgrounds, which lack the *smp1*^*R110Q,P269Q*^ and *hot1*^*P107S,H274Y*^ alleles, the *gpd1*^*L164P*^ allele had a clear reducing effect on glycerol yield compared to the wild type *GPD1* allele (Figure 6). On the other hand, its expression in the strains 26B and 26B/ER7A, which contain the *smp1*^*R110Q,P269Q*^ and *hot1*^*P107S,H274Y*^ alleles, resulted in a similar glycerol yield as the wild type *GPD1* allele. This suggests that the *smp1*^*R110Q,P269Q*^ and *hot1*^*P107S,H274Y*^ alleles and/or other alleles present in the superior strains suppress the effect of the *gpd1*^*L164P*^ allele. The epistatic effect may be explained at the biochemical level by the fact that the reduction in expression of *GPD1*, caused by the *smp1*^*R110Q,P269Q*^ and *hot1*^*P107S,H274Y*^ alleles, is so strong that the mutation in *GPD1* itself has no significant effect anymore. Hot1 activates transcription of *GPD1* and other HOG-dependent genes under osmostress [31,32,38]. Alepuz et al. [31] proposed that Hot1 serves as an anchor for Hog1, which directly recruits the RNA polymerase II complex. The position of the Hog1 binding domain in Hot1 is unknown. It is unclear how the two mutations in the 26B allele, *hot1*^*P107S,H274Y*^, could affect the functioning of the protein.

Interestingly, although all mutant genes revealed in this work had already been identified previously using classical molecular genetics approaches, our work clearly indicates the potential of complex trait analysis for identifying new alleles of known components, and possibly also completely new components, in signaling pathways and other cellular functions. In our case, it seems plausible that continuation of backcrossing with new segregants from subsequent generations, displaying low glycerol/high ethanol yield and lacking (most of) the previously identified alleles, might reveal new players in the HOG signaling pathway or in transcriptional control of *GPD1*.

## Conclusions

This work has shown that yeast biodiversity harbors multiple mutant alleles of genes in glycerol biosynthesis and its regulation that can be used to lower the glycerol yield in bioethanol production. Since these are natural alleles that cause subtle changes and that act at different levels in glycerol biosynthesis and its regulation, their use minimizes the risk of negative side-effects on other industrially important properties, as is often seen with drastic alterations of structural and regulatory genes by genetic modification.

## Materials and methods

### Microbial strains, cultivation conditions and plasmids

All *S. cerevisiae* strains used are listed in Table 1. Yeast strains were grown in 1% yeast extract, 2% peptone media (YP) with glucose as carbon source in the indicated concentration. *E. coli* strain DH5α^TM^ (Invitrogen Corp., Carlsbad) was used for amplification of plasmids. The strain was grown in Luria-Bertani (LB) medium containing 0.5% (w/v) yeast extract, 1% (w/v) Bacto tryptone, 1% (w/v) NaCl, (pH 7.5) at 37°C. *E. coli* transformation and isolation of plasmid DNA was carried out using standard techniques [39]. Transformants were selected on LB medium containing 100 μg/ml ampicillin. The plasmids used are shown in Table 2.

**Table 1:** ***Saccharomyces cerevisiae *strains used**

**Strain**	**Genotype**	**Source**
CBS6412	Diploid*, ssk1*^*E330N…K356N*^*/ssk1*^*E330N…K356N*^	CBS-KNAW
Ethanol Red	Diploid*, SSK1/SSK1*	Fermentis, S. I. Lesaffre
ER7A	Segregant 7A of Ethanol Red, *Mat****α***	This study
CBS4C	Segregant 4C of CBS6412, *Mat****a,****ssk1*^*E330N…K356N*^	This study
26B	Segregant of the cross ER7A x CBS4C, *Mat****α,****SSK1*	This study
26B Mat**a**	Mating type switch of 26B to *Mat****a***	This study
26B/ER7A	Hybrid diploid 26B/ER7A	This study
26B smp1∆/ER7A	Hybrid diploid 26B smp1∆/ER7A	This study
26B/ER7A smp1∆	Hybrid diploid 26B/ER7A smp1∆	This study
26B gpd1∆/ER7A	Hybrid diploid 26B gpd1∆/ER7A	This study
26B/ER7A gpd1∆	Hybrid diploid 26B/ER7A gpd1∆	This study
26B hot1∆/ER7A	Hybrid diploid 26B hot1∆/ER7A	This study
26B/ER7A hot1∆	Hybrid diploid 26B/ER7A hot1∆	This study
BY4742 gpd1∆ YCplac33	Haploid*, gpd1∆*, YCplac33	This study
BY4742 gpd1∆ YCplac33 GPD1-ER7A	Haploid, *gpd1∆*, YCplac33 *GPD1-ER7A*	This study
BY4742 gpd1∆ YCplac33 gpd1^L164P^-CBS4C	Haploid, *gpd1∆*, YCplac33 *gpd1*^*L164P*^*-CBS4C*	This study
26B gpd1∆ YCplac33	Haploid, *ura3∆, gpd1∆*, YCplac33	This study
26B gpd1∆ YCplac33 GPD1-ER7A	Haploid, *ura3∆, gpd1∆*, YCplac33 *GPD1-ER7A*	This study
26B gpd1∆ YCplac33 gpd1^L164P^-CBS4C	Haploid, *ura3∆, gpd1∆*, YCplac33 *gpd1*^*L164P*^*-CBS4C*	This study
ER7A gpd1∆ YCplac33	Haploid, *ura3∆, gpd1∆*, YCplac33	This study
ER7A gpd1∆ YCplac33 GPD1-ER7A	Haploid, *ura3∆, gpd1∆*, YCplac33 *GPD1-ER7A*	This study
ER7A gpd1∆ YCplac33 gpd1^L164P^-CBS4C	Haploid, *ura3∆, gpd1∆*, YCplac33 *gpd1*^*L164P*^*-CBS4C*	This study
26B/ER7A gpd1∆/∆ YCplac33	Diploid, *ura3∆/*∆*, gpd1∆/*∆, YCplac33	This study
26B/ER7A gpd1∆/∆ YCplac33 GPD1-ER7A	Diploid, *ura3∆/*∆*, gpd1∆/*∆, YCplac33 *GPD1-ER7A*	This study
26B/ER7A gpd1∆/∆ YCplac33 gpd1^L164P^-CBS4C	Diploid, *ura3∆/*∆*, gpd1∆/*∆, YCplac33 *gpd1*^*L164P*^*-CBS4C*	This study

**Table 2:** Plasmids used

**Plasmid**	**Description**	**Reference**
pUG6	*E. coli*/vector containing, Amp^+^, *loxP-KanMX6-loxP* disruption cassette	[46]
pUG66	*E. coli*/vector containing, Amp^+^, *loxP-ble*^*R*^*-loxP* disruption cassette	[46]
pFL39 GAL1 HO KanMX	*vector containing HO gene*	Lab stock
YCplac33	*yeast shuttle vector, URA3*	Lab stock
YCplac33/GPD1-ER7A	*yeast shuttle vector, URA3 GPD1-ER7A*	This work
YCplac33/gpd1^L164P^-CBS4C	*yeast shuttle vector URA3, gpd1*^*L164P*^	This work

### Mating, mating type switch, sporulation and internal crosses

Mating and sporulation were carried out according to standard procedures [40]. Mating type of segregants was determined by diagnostic PCR for the *MAT* locus [41]. Mating type switching was performed by induction of the HO-gene expressed from the plasmid pFL39 *GAL1 HO KanMX*. Meiotic spores of the hybrid 26B/ER7A were isolated by random spore analysis [42]. The hybrid 26B/ER7A was plated on a sporulation plate for a period of about two weeks up to 1 month until asci were observed. The yeast cells were washed off the sporulation plate and suspended in 25 ml of MilliQ water, in a 300 ml Erlenmeyer flask together with sterile 0.45 mm glass beads. 500 μl Zymolyase (10 mg/ml) and 10 μl of β-mercaptoethanol were added to the cell suspension in order to degrade the asci. This cell suspension was incubated overnight at 30°C by shaking at 200 rpm. The cell suspension was transferred to a 50 ml tube together with the glass beads, and shaken vigorously. The cell debris was centrifuged at 20000 rpm for 20 min. The supernatant was discarded and the pellet was suspended in 5 ml of Nonidet P-40 and placed on ice for 15 min. This was followed by 4 rounds of sonication (30 s, 75%). The cell suspension rested 2 min on ice between two rounds. After that, the cell suspension was centrifuged for 10 min at 3000 rpm, the supernatant was discarded, and the cells were re-suspended in 1.5% Nonidet P-40. This procedure was repeated once, followed by 4 more rounds of sonication and incubation on ice. Lastly, the cell suspension was centrifuged for 10 min at 3000 rpm. The supernatant was discarded and cells were re-suspended in 300 ml of MilliQ water. The cell suspension was diluted to obtain single colonies on plates. Plates were incubated at 30°C until single colonies were visible. These single colonies were re-plated and checked for mating type to confirm haploidy. Usually, this procedure yielded 90% haploids. Crossing of 26B and ER7A was carried out as follows. First, diploids were isolated from the cross of ER7A and 26B. For this purpose, the mating type of single colonies resulting from the cross between ER7A and 26B was checked. In the first step of internal crossing, the diploids were incubated on a sporulation plate until sufficient asci were visible. In the second step, spores were isolated using random spore analysis (see further). The isolated spores were all plated on YD and incubated for 2 days at 30°C to ensure that enough diploids had been formed. In the last step, newly formed diploids were transferred to a new sporulation plate to start the next cycle of internal crossing.

### Fermentation conditions

The 26B, ER7A and CBS4C strains were tested in two fermentations: minimal medium and YP 10% glucose. The minimal medium was composed of 1.9 g l^-1^ yeast nitrogen base (Difco), 5 g l^-1^ ammonium sulphate, 250 mg l^-1^ leucine, 50 mg l^-1^ uracil, 100 mg l^-1^ histidine, 30 mg l^-1^ lysine, 20 mg l^-1^ methionine and 50 g l^-1^ glucose. The inoculum culture was grown overnight in minimal medium containing 2% [w/v] glucose and was used for inoculation of the fermentation medium at an initial OD of 1. Fermentations were carried out in Erlenmeyer flasks, which were equipped with air locks, ensuring the exclusion of oxygen but allowing the release of CO_2_. The fermentations were performed at 30°C and cultures were continuously stirred at 200 rpm.

The parental strains, ER7A and 26B, as well as CBS4C, were additionally tested in YPD, which contained 0.2% [w/v] yeast extract, 0.6% [w/v] peptone, and 10% [w/v] glucose, to mimick the free amino nitrogen content present in wheat liquefact. The fermentations were carried out in cylindrical glass tubes, which were closed with a rubber stopper containing a glass pipe, sealed off with a cotton plug to release CO_2_. 100 ml fermentation medium was added to each tube. Inoculum cultures were grown statically overnight at 30°C in 5 ml of YD medium one day ahead of the fermentation, and this culture was used completely to inoculate the 100 ml fermentation tube. The empty weight, starting weight and weight after 72 h fermentation of the tubes was measured to determine the net weight loss of the medium during the fermentation. Fermentations ran for 72 h at 30°C and were stirred at 200 rpm. After 72 h, the final weight was determined. The fermentation broth was cooled for 1 night at 4°C prior to the analysis of glycerol and ethanol concentrations, in order to minimize evaporation of ethanol during the sample taking. The fermentations of the initial screen of 26B/ER7A segregants, those of the reciprocal hemizygosity analysis, and those of the *gpd1*Δ strains were carried out in 100 ml YP 10% glucose.

Screening of additional selected segregants was downscaled to 5 ml fermentation cultures. The pre-culture was started one day ahead of the fermentation in 3 ml of YD medium. Cultures were grown statically overnight at 30°C. The next day, the pre-cultures were used to inoculate the 5 ml fermentation in a proportion of 1/20. Fermentations were kept for 96 h at 30°C and afterwards placed at 4°C overnight prior to the analysis of the fermentation broth in order to prevent ethanol evaporation.

### Determination of fermentation parameters

In all fermentations weight loss was used to follow the progress of the fermentation. Glucose, glycerol and ethanol in the medium were determined by HPLC (Waters® isocratic Breeze^TM^ HPLC, ion exchange column WAT010290). Column temperature was 75°C, 5 mM H_2_SO_4_ was used as eluent with a flow rate of 1 ml min^-1^ and refractive index detection was used (Waters, 2414 RI detector). The product yield was calculated from the final product concentration (g. l^-1^) and the difference in glucose concentration at the start and end of the fermentation (consumed glucose in g. l^-1^). Yields of strains used for screening, RHA and the *gpd*1∆ complementation analysis were related to the yield of 26B in the same experiment in order to decrease variance between different experiments.

Yields were calculated with the following formulas:

Absolute yield: $Y_{S/i=}\frac{c_{i}^{\mathrm{final}}}{\left(c_{s}^{\mathrm{ini}}-c_{S}^{\mathrm{final}}\right)}$

Relative yield: $Y_{\%}=\frac{Y_{S/i}^{\mathrm{segr}}}{Y_{S/i}^{26B}}$

### DNA methods

Yeast genomic DNA was extracted with Phenol/Chloroform/Isoamyl-alcohol (25:24:1) [43] and further purified with diethyl-ether extraction or ethanol precipitation if required. PCR was performed with high-fidelity polymerases Phusion^TM^ (Finnzymes) or ExTaq^TM^ (TaKaRa) for cloning and amplification of deletion or insertion cassettes, and sequencing purposes. Sequencing was carried out using the dideoxy chain-termination method [44] at the VIB Genetic Service Facility (Antwerp). The sequences were analyzed with geneious (Geneious Basic 5.3.4), SeqMan (Lasergene Coresuite 8) or CLC DNA workbench (CLC bio) software.

### Pooled-segregant whole-genome sequence analysis

The segregant 26 was isolated from the cross of CBS4C and ER7A. This segregant was backcrossed with its own parent ER7A. From this backcross, the 22 most superior segregants (lowest glycerol production) were assembled in the ‘selected pool’ while 22 random segregants were used to assemble the ‘unselected pool’. The two pools were made by combining equal amounts of cells based on OD_600_. High molecular weight DNA (3 μg, ~ 20kb fragments) was isolated from the pools and parent strains according to Johnston and Aust [45]. The purity of the DNA sample was estimated from UV measurement (260/280 = 1.7-2.0). The DNA samples were provided to BGI (Hong Kong, China) for whole-genome sequence analysis by Illumina technology.

Mapping of short read sequences, variant calling and QTL analysis were carried out as described earlier by Swinnen et al. [20] and by Hubmann et al. [19]. The SNP variant frequencies were calculated by dividing the number of the alternative variant by the total number of aligned reads. A very high or a very low frequency was a sign of a one-sided SNP segregation preferentially coming from one parent, indicating a genetic linkage to the trait of interest. Genetic linkage was statistically confirmed using EXPloRA (Duitama et al. in preparation) or the methods described earlier [20].

### Detection of SNP markers

Individual SNPs were scored by PCR. The forward and reverse primer contained the nucleotide of ER7A or CBS4C as the 3’ terminal nucleotide, respectively. The annealing temperature was optimized using DNA of ER7A and CBS4C so as to allow only hybridization with primers containing an exact match.

### Reciprocal hemizygosity analysis (RHA)

For RHA analysis [25], two diploid strains were constructed by crossing 26B and ER7A wild type or deletion strains for the candidate gene, so that the resulting diploids only contained a single allele from either 26B or ER7A for the candidate gene being evaluated. Deletion cassettes for *SMP1*, *HOT1* and *GPD1* were constructed as described by Gueldner et al. [46] using the phleomycin resistance marker *ble*^*R*^. After the transformation, the gene deletions were verified by PCR. RHA was performed with three independent isolates of all tested diploids.

### Construction of *YCplac GPD1* plasmids

The primers, A-3709 and A-3743, were used to PCR amplify genomic DNA of CBS4C and ER7A chr. IV (410523 – 413479), containing the promoter, *GPD1* ORF, and terminator. The resulting PCR fragment was digested with *KpnI,* purified, and ligated to the plasmid *YCplac33*, which was digested with *KpnI* prior to ligation. The constructs *YCplac33 GPD1-ER7A* and YCplac33 *gpd1*^*L164P*^*-CBS4C* were verified using Sanger sequencing. *URA3* was deleted in the strain 26B *gpd1∆* and ER7A *gpd1∆*. Both strains were mated to obtain the diploid strain ER7A/26B *gpd1∆/∆ ura3∆/∆*. The deletion cassette was constructed as described by Gueldner et al. [46] with the geneticin resistance marker *KanMX6.* Transformants were selected simultaneously for the two selectable markers on phleomycin and geneticin, to avoid a cassette switch. *URA3* gene deletion was confirmed by PCR and absence of growth on SD-ura plates. YCplac33 *GPD1-ER7A,* YCplac33 *gpd1*^*L164P*^*-CBS4C* and the empty plasmid were transferred to the strain BY4742 *gpd1∆,* 26B *gpd1∆ ura3∆*, ER7A *gpd1∆ ura3∆*, and ER7A/26B *gpd1∆/∆ ura3∆/∆*, using the LiAc/PEG transformation method [47].

### Data deposition

Sequencing data have been deposited at the SRA database (NCBI), http://www.ncbi.nlm.nih.gov/sra, with the account number SRA059109.

## Abbreviations

OD: Optical density; YP: Yeast extract peptone; YPD: Yeast extract peptone dextrose; SD: Synthetic dextrose; LB: Luria-Bertani; PCR: Polymerase chain reaction; HPLC: High performance liquid chromatography; QTL: Quantitative trait locus; QTLs: Quantitative trait loci; SNP: Single nucleotide polymorphism; chr: chromosome; F1: First generation; F5: Fifth generation; RHA: Reciprocal hemizygosity analysis; GPDH: Glycerol-3-phosphate dehydrogenase; DHAP: Dihydroxyacetone phosphate; HOG: Pathway high osmolarity glycerol pathway; w/v: weight/volume; rpm: rounds per minute.

## Competing interests

The authors declare that they have no conflict of interest. The use of the mutant alleles identified in this paper for reduction of the glycerol/ethanol ratio in fermentations with industrial yeast strains will be protected by a patent application.

## Authors’ contributions

GH, MF, EN and JMT designed the experiments, GH and LM performed the experiments, GH, LM, MF and JMT analyzed the data, JD provided bioinformatics analysis and GH, MF, EN and JMT wrote the paper. All authors read and approved the final manuscript.

## References

[B1] Remize F Roustan JL Sablayrolles JM Barre P Dequin S Glycerol overproduction by engineered *Saccharomyces cerevisiae* wine yeast strains leads to substantial changes in by-product formation and to a stimulation of fermentation rate in stationary phase Appl Environ Microbiol 1999 65 143 149 9872772 10.1128/aem.65.1.143-149.1999 PMC90995

[B2] Hubmann G Guillouet S Nevoigt E Gpd1 and Gpd2 fine-tuning for sustainable reduction of glycerol formation in *Saccharomyces cerevisiae* Appl Environ Microbiol 2011 77 5857 5867 10.1128/AEM.05338-11 21724879 PMC3165387

[B3] Ansell R Granath K Hohmann S Thevelein JM Adler L The two isoenzymes for yeast NAD + −dependent glycerol 3-phosphate dehydrogenase encoded by *GPD1* and *GPD2* have distinct roles in osmoadaptation and redox regulation EMBO J 1997 16 2179 2187 10.1093/emboj/16.9.2179 9171333 PMC1169820

[B4] Bjorkqvist S Ansell R Adler L Liden G Physiological response to anaerobicity of glycerol-3-phosphate dehydrogenase mutants of *Saccharomyces cerevisiae* Appl Environ Microbiol 1997 63 128 132 8979347 10.1128/aem.63.1.128-132.1997 PMC168310

[B5] Nissen TL Hamann CW Kielland-Brandt MC Nielsen J Villadsen J Anaerobic and aerobic batch cultivations of *Saccharomyces cerevisiae* mutants impaired in glycerol synthesis Yeast 2000 16 463 474 10.1002/(SICI)1097-0061(20000330)16:5<463::AID-YEA535>3.0.CO;2-3 10705374

[B6] van Maris AJ Geertman JM Vermeulen A Groothuizen MK Winkler AA Piper MD van Dijken JP Pronk JT Directed evolution of pyruvate decarboxylase-negative *Saccharomyces cerevisiae*, yielding a C2-independent, glucose-tolerant, and pyruvate-hyperproducing yeast Appl Environ Microbiol 2004 70 159 166 10.1128/AEM.70.1.159-166.2004 14711638 PMC321313

[B7] Hong KK Vongsangnak W Vemuri GN Nielsen J Unravelling evolutionary strategies of yeast for improving galactose utilization through integrated systems level analysis Proc Natl Acad Sci USA 2011 108 12179 12184 10.1073/pnas.1103219108 21715660 PMC3141923

[B8] Hong KK Nielsen J Recovery of phenotypes obtained by adaptive evolution through inverse metabolic engineering Appl Environ Microbiol 2012 78 7579 7586 10.1128/AEM.01444-12 22904057 PMC3485731

[B9] Bro C Regenberg B Forster J Nielsen J In silico aided metabolic engineering of *Saccharomyces cerevisiae* for improved bioethanol production Metab Eng 2006 8 102 111 10.1016/j.ymben.2005.09.007 16289778

[B10] Basso LC de Amorim HV de Oliveira AJ Lopes ML Yeast selection for fuel ethanol production in Brazil FEMS Yeast Res 2008 8 1155 1163 10.1111/j.1567-1364.2008.00428.x 18752628

[B11] Remize F Cambon B Barnavon L Dequin S Glycerol formation during wine fermentation is mainly linked to Gpd1p and is only partially controlled by the HOG pathway Yeast 2003 20 1243 1253 10.1002/yea.1041 14618562

[B12] Styger G Prior B Bauer FF Wine flavor and aroma J Ind Microbiol Biotechnol 2011 38 1145 1159 10.1007/s10295-011-1018-4 21786136

[B13] Schuller D Casal M The use of genetically modified *Saccharomyces cerevisiae* strains in the wine industry Appl Microbiol Biotechnol 2005 68 292 304 10.1007/s00253-005-1994-2 15856224

[B14] Schmidtke LM Blackman JW Agboola SO Production technologies for reduced alcoholic wines J Food Sci 2012 77 R25 R41 10.1111/j.1750-3841.2011.02448.x 22260123

[B15] Larsson K Ansell R Eriksson P Adler L A gene encoding sn-glycerol 3-phosphate dehydrogenase (NAD+) complements an osmosensitive mutant of *Saccharomyces cerevisiae* Mol Microbiol 1993 10 1101 1111 10.1111/j.1365-2958.1993.tb00980.x 7934860

[B16] Albertyn J Hohmann S Thevelein JM Prior BA GPD1, which encodes glycerol-3-phosphate dehydrogenase, is essential for growth under osmotic stress in *Saccharomyces cerevisiae*, and its expression is regulated by the high-osmolarity glycerol response pathway Mol Cell Biol 1994 14 4135 4144 8196651 10.1128/mcb.14.6.4135-4144.1994 PMC358779

[B17] Pahlman AK Granath K Ansell R Hohmann S Adler L The yeast glycerol 3-phosphatases Gpp1p and Gpp2p are required for glycerol biosynthesis and differentially involved in the cellular responses to osmotic, anaerobic, and oxidative stress J Biol Chem 2001 276 3555 3563 10.1074/jbc.M007164200 11058591

[B18] Hohmann S Osmotic stress signaling and osmoadaptation in yeasts Microbiol Mol Biol Rev 2002 66 300 372 10.1128/MMBR.66.2.300-372.2002 12040128 PMC120784

[B19] Hubmann G Foulquié-Moreno MR Nevoigt E Duitama J Meurens N Pais TM Mathé L Saerens S Nguyen HTT Swinnen S Quantitative trait analysis of yeast biodiversity yields novel gene tools for metabolic engineering Metab Eng 2013 In press 10.1016/j.ymben.2013.02.006 23518242

[B20] Swinnen S Schaerlaekens K Pais T Claesen J Hubmann G Yang Y Demeke M Foulquie-Moreno MR Goovaerts A Souvereyns K Identification of novel causative genes determining the complex trait of high ethanol tolerance in yeast using pooled-segregant whole-genome sequence analysis Genome Res 2012 22 975 984 10.1101/gr.131698.111 22399573 PMC3337442

[B21] Swinnen S Thevelein JM Nevoigt E Genetic mapping of quantitative phenotypic traits in *Saccharomyces cerevisiae* FEMS Yeast Res 2012 12 215 227 10.1111/j.1567-1364.2011.00777.x 22150948

[B22] Ehrenreich IM Torabi N Jia Y Kent J Martis S Shapiro JA Gresham D Caudy AA Kruglyak L Dissection of genetically complex traits with extremely large pools of yeast segregants Nature 2010 464 1039 1042 10.1038/nature08923 20393561 PMC2862354

[B23] Parts L Cubillos FA Warringer J Jain K Salinas F Bumpstead SJ Molin M Zia A Simpson JT Quail MA Revealing the genetic structure of a trait by sequencing a population under selection Genome Res 2011 21 1131 1138 10.1101/gr.116731.110 21422276 PMC3129255

[B24] Liti G Louis EJ Advances in quantitative trait analysis in yeast PLoS Genet 2012 8 e1002912 10.1371/journal.pgen.1002912 22916041 PMC3420948

[B25] Steinmetz LM Sinha H Richards DR Spiegelman JI Oefner PJ McCusker JH Davis RW Dissecting the architecture of a quantitative trait locus in yeast Nature 2002 416 326 330 10.1038/416326a 11907579

[B26] Lorenz K Cohen BA Small- and large-effect quantitative trait locus interactions underlie variation in yeast sporulation efficiency Genetics 2012 192 1123 1132 10.1534/genetics.112.143107 22942125 PMC3522155

[B27] Sinha H David L Pascon RC Clauder-Munster S Krishnakumar S Nguyen M Shi G Dean J Davis RW Oefner PJ Sequential elimination of major-effect contributors identifies additional quantitative trait loci conditioning high-temperature growth in yeast Genetics 2008 180 1661 1670 10.1534/genetics.108.092932 18780730 PMC2581965

[B28] Demogines A Smith E Kruglyak L Alani E Identification and dissection of a complex DNA repair sensitivity phenotype in Baker’s yeast PLoS Genet 2008 4 e1000123 10.1371/journal.pgen.1000123 18617998 PMC2440805

[B29] Claesen J Clement L Shkedy Z Foulquié-Moreno MR Burzykowski T Simultaneous mapping of multiple gene loci with pooled segregants PLoS One 2013 In press 10.1371/journal.pone.0055133 PMC3575411 23441149

[B30] de Nadal E Casadome L Posas F Targeting the MEF2-like transcription factor Smp1 by the stress-activated Hog1 mitogen-activated protein kinase Mol Cell Biol 2003 23 229 237 10.1128/MCB.23.1.229-237.2003 12482976 PMC140668

[B31] Alepuz PM de Nadal E Zapater M Ammerer G Posas F Osmostress-induced transcription by Hot1 depends on a Hog1-mediated recruitment of the RNA Pol II EMBO J 2003 22 2433 2442 10.1093/emboj/cdg243 12743037 PMC156001

[B32] Rep M Reiser V Gartner U Thevelein JM Hohmann S Ammerer G Ruis H Osmotic stress-induced gene expression in *Saccharomyces cerevisiae* requires Msn1p and the novel nuclear factor Hot1p Mol Cell Biol 1999 19 5474 5485 10409737 10.1128/mcb.19.8.5474 PMC84389

[B33] Giaever G Chu AM Ni L Connelly C Riles L Veronneau S Dow S Lucau-Danila A Anderson K Andre B Functional profiling of the *Saccharomyces cerevisiae* genome Nature 2002 418 387 391 10.1038/nature00935 12140549

[B34] Winzeler EA Shoemaker DD Astromoff A Liang H Anderson K Andre B Bangham R Benito R Boeke JD Bussey H Functional characterization of the *S. cerevisiae* genome by gene deletion and parallel analysis Science 1999 285 901 906 10.1126/science.285.5429.901 10436161

[B35] Beavis W Paterson AH QTL analyses: Power, precision and accuracy Molecular Dissection of Complex Traits 1998 Boca Raton, FL, USA: CRC Press Inc 145 161

[B36] Dodou E Treisman R The *Saccharomyces cerevisiae* MADS-box transcription factor Rlm1 is a target for the Mpk1 mitogen-activated protein kinase pathway Mol Cell Biol 1997 17 1848 1859 9121433 10.1128/mcb.17.4.1848 PMC232032

[B37] Marchler-Bauer A Anderson JB Chitsaz F Derbyshire MK DeWeese-Scott C Fong JH Geer LY Geer RC Gonzales NR Gwadz M CDD: specific functional annotation with the conserved domain database Nucleic Acids Res 2009 37 D205 210 10.1093/nar/gkn845 18984618 PMC2686570

[B38] Rep M Krantz M Thevelein JM Hohmann S The transcriptional response of *Saccharomyces cerevisiae* to osmotic shock. Hot1p and Msn2p/Msn4p are required for the induction of subsets of high osmolarity glycerol pathway-dependent genes J Biol Chem 2000 275 8290 8300 10.1074/jbc.275.12.8290 10722658

[B39] Sambrook J Maniatis T Fritsch EF Molecular cloning : a laboratory manual 1989 2 Cold Spring Harbor, N.Y.: Cold Spring Harbor Laboratory

[B40] Sherman F Hicks J Micromanipulation and dissection of asci Methods Enzymol 1991 194 21 37 2005789 10.1016/0076-6879(91)94005-w

[B41] Huxley C Green ED Dunham I Rapid assessment of S. cerevisiae mating type by PCR Trends Genet 1990 6 236 2238077 10.1016/0168-9525(90)90190-h

[B42] Treco DA Winston F Growth and manipulation of yeast Curr Protoc Mol Biol 2001 Unit13 12 Volume Chapter 13. 2008/02/12 edition 10.1002/0471142727.mb1302s19 18265098

[B43] Hoffman CS Winston F A ten-minute DNA preparation from yeast efficiently releases autonomous plasmids for transformation of *Escherichia coli* Gene 1987 57 267 272 10.1016/0378-1119(87)90131-4 3319781

[B44] Sanger F Coulson AR A rapid method for determining sequences in DNA by primed synthesis with DNA polymerase J Mol Biol 1975 94 441 448 10.1016/0022-2836(75)90213-2 1100841

[B45] Johnston CG Aust SD Detection of *Phanerochaete chrysosporium* in soil by PCR and restriction enzyme analysis Appl Environ Microbiol 1994 60 2350 2354 8074515 10.1128/aem.60.7.2350-2354.1994 PMC201654

[B46] Gueldener U Heinisch J Koehler GJ Voss D Hegemann JH A second set of loxP marker cassettes for Cre-mediated multiple gene knockouts in budding yeast Nucleic Acids Res 2002 30 e23 10.1093/nar/30.6.e23 11884642 PMC101367

[B47] Gietz D St Jean A Woods RA Schiestl RH Improved method for high efficiency transformation of intact yeast cells Nucleic Acids Res 1992 20 1425 10.1093/nar/20.6.1425 1561104 PMC312198

